# High resolution fluorescence imaging of the alveolar scaffold as a novel tool to assess lung injury

**DOI:** 10.1038/s41598-024-57313-6

**Published:** 2024-03-20

**Authors:** Sandra Lindstedt, Qi Wang, Anna Niroomand, Martin Stenlo, Snejana Hyllen, Leif Pierre, Franziska Olm, Nicholas B. Bechet

**Affiliations:** 1 Wallenberg Centre for Molecular Medicine, Lund University, Lund, Sweden; 2 Department of Clinical Sciences, Lund University, Lund, Sweden; 3 Lund Stem Cell Centre, Lund University, Lund, Sweden; 4 Department of Cardiothoracic Surgery and Transplantation, Skåne University Hospital, Lund, Sweden; 5 Department of Cardiothoracic Anaesthesia and Intensive Care, Skåne University Hospital, Lund, Sweden

**Keywords:** Biological techniques, Imaging, Fluorescence imaging

## Abstract

Acute lung injury (ALI) represents an aetiologically diverse form of pulmonary damage. Part of the assessment and diagnosis of ALI depends on skilled observer-based scoring of brightfield microscopy tissue sections. Although this readout is sufficient to determine gross alterations in tissue structure, its categorical scores lack the sensitivity to describe more subtle changes in lung morphology. To generate a more sensitive readout of alveolar perturbation we carried out high resolution immunofluorescence imaging on 200 μm lung vibratome sections from baseline and acutely injured porcine lung tissue, stained with a tomato lectin, Lycopersicon Esculentum Dylight-488. With the ability to resolve individual alveoli along with their inner and outer wall we generated continuous readouts of alveolar wall thickness and circularity. From 212 alveoli traced from 10 baseline lung samples we established normal distributions for alveolar wall thickness (27.37; 95% CI [26.48:28.26]) and circularity (0.8609; 95% CI [0.8482:0.8667]) in healthy tissue. Compared to acutely injured lung tissue baseline tissue exhibited a significantly lower wall thickness (26.86 ± 0.4998 vs 50.55 ± 4.468; p = 0.0003) and higher degree of circularityϕ≤ (0.8783 ± 0.01965 vs 0.4133 ± 0.04366; p < 0.0001). These two components were subsequently combined into a single more sensitive variable, termed the morphological quotient (MQ), which exhibited a significant negative correlation (R^2^ = 0.9919, p < 0.0001) with the gold standard of observer-based scoring. Through the utilisation of advanced light imaging we show it is possible to generate sensitive continuous datasets describing fundamental morphological changes that arise in acute lung injury. These data represent valuable new analytical tools that can be used to precisely benchmark changes in alveolar morphology both in disease/injury as well as in response to treatment/therapy.

## Introduction

Acute lung injury (ALI) is a form of respiratory failure in which acute inflammation yields disturbances in the endothelial and epithelial barriers, and in its most severe form, manifests clinically as acute respiratory distress syndrome (ARDS)^1–3^. Rather than a specific disorder with a precise aetiology, ALI represents a diverse manifestation of bronchoalveolar perturbations. The gold-standard for histopathologic assessment of ALI rests in observer-based scoring of haematoxylin and eosin (H&E) stained sections where injured tissue exhibits infiltration with immune cells in the alveolar wall and space, thickening of the alveolar wall, the aggregation of proteinaceous debris, haemorrhage, and the formation of hyaline membranes^2^. While well-defined clinically, the validation of ALI in animal models is more controversial^3–5^. To ensure better standardisation in animal models, criteria detailing the main features of experimental injury have been defined, including substantiating alterations in the alveolar-capillary barrier, inflammatory infiltration, physiological dysfunction and finally, histologic evidence of injury^6,7^.

For histological evidence of injury, the sub-criteria included also rely on dye-based (H&E) stains and immunohistochemical (IHC) techniques that utilise bright field microscopy, with observer-based scoring ^6,7^. As similar processing and scoring is carried out in the clinic, it is important to maintain these readouts in animal models to make more reliable inferences that translate from the model to the patient^5^. However, the main drawbacks of these readouts, both human tissue and animal models, is that they require trained scorers, and that the scoring outcomes are categorical and thus lack the sensitivity to detect more subtle morphological changes. If we wish to explore more subtle changes and better understand the molecular mechanisms of a given ALI model or patient cohort, which may be more productive in developing and guiding therapeutic interventions, it is pivotal that we work towards generating more sensitive and detailed readouts that make full use of the technology available.

One such rapidly expanding technology that can be leveraged in this regard is immunofluorescence (IF) imaging in conjunction with advanced light microscopy. The analysis pipelines that lend themselves to this type of imaging data afford more objective evaluations that generate continuous rather than categorical data sets. These readouts have the capacity to uncover more subtle changes in bronchoalveolar morphology that may go unnoticed by the eye of human observers/scorers.

While multiplex IF and advanced light imaging panels are well established in rodent models these pipelines have been less well developed in large animals^8–10^. Yet, large animal models are a critical step in translating findings to the clinic, highlighting the importance of developing more advanced readouts in these species^11–13^.

To this end we utilised a simple fluorescent-based imaging approach by exploiting a lectin-fluorophore conjugate, Lycopersicon Esculentum lectin (LEA), which exhibits a strong affinity for bronchoalveolar epithelial cells^14^. While LEA has been widely utilised in neuroscience and other vascular research to visualise blood vessels via interactions with endothelial cell surface carbohydrates, it has been less well exploited in pulmonary research^15–17^. Herein we describe a fluorescent LEA-based pulmonary staining approach applied to free floating lung sections. High resolution confocal microscopy of individual alveoli generated continuous data sets detailing alveolar wall thickness and circularity, both of which are affected in acute lung injury, and represent a more sensitive readout of alveolar perturbation.

## Results

### Lycopersicon Esculentum lectin exposes the alveolar scaffold

**Figure 1:**
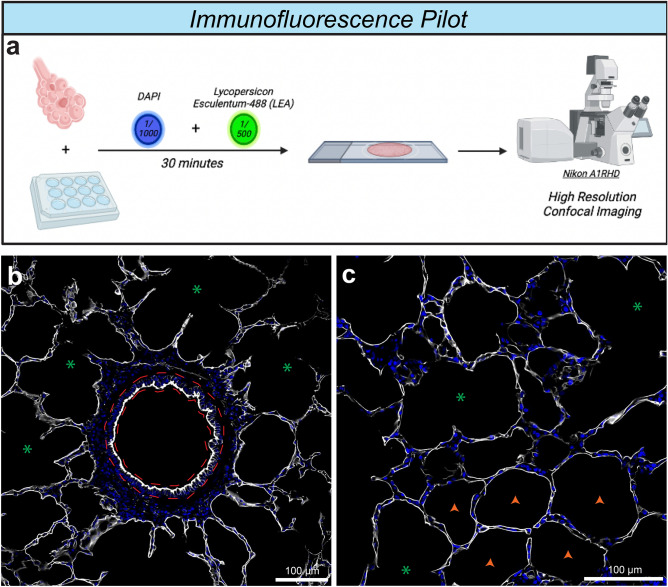
(**a**) Schematic overview of tissue staining and imaging pipeline utilised to investigate alveolar structure. (**b**) LEA staining showing binding to bronchial (red outline) and terminal bronchiolar epithelium (green asterisks). (**c**) LEA staining showing binding terminal bronchiolar epithelium (green asterisks) and alveolar epithelium (orange arrowheads).

To test whether Lycopersicon Esculentum Dylight-488 (LEA-488) could generate a robust epithelial-based signal in lung tissue, we incubated 200 μm free floating sections, generated as previously decrsibed^18^, in PBS with DAPI and LEA-488 (Fig. 1a). Following a 30-min incubation, lung sections were mounted and imaged with a confocal microscope (Fig. 1a).

**Figure 2:**
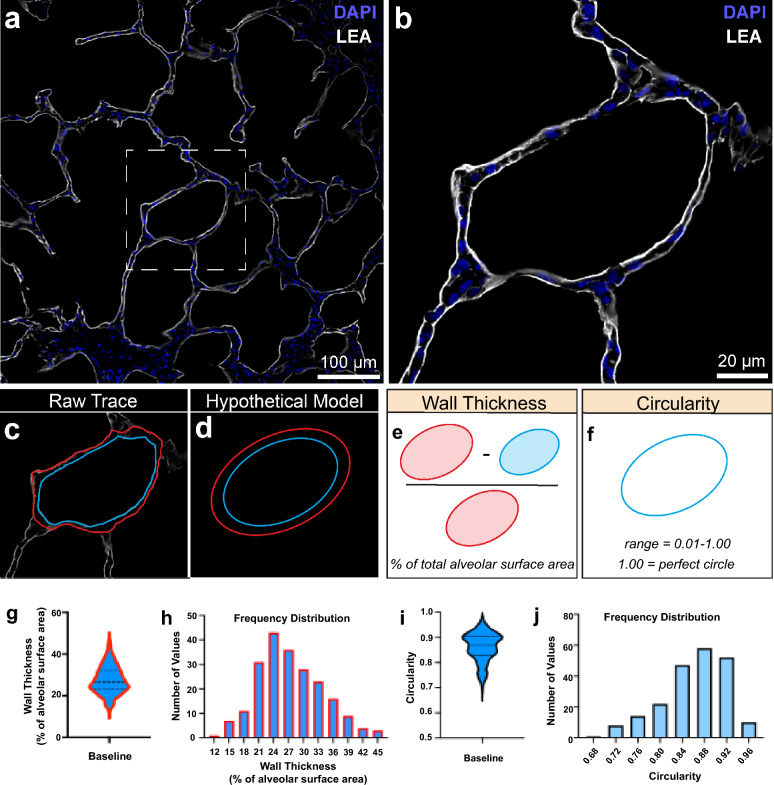
(**a**) Representative image of Lycopersicon Esculentum lectin (LEA) and DAPI fluorescent stain of alveoli and terminal bronchioles. (**b**) Representative high-resolution image of single alveolus from (**a**). (**c**) Raw trace from image processing software of inner and outer alveolar wall based on LEA signal. (**d**) Hypothetical representation of traces from (**c**). (**e**) Schematic showing how alveolar wall thickness is calculated. **f**) Schematic illustrating concept of circularity. (**g**) Absolute distribution of alveolar wall thickness from cohort of healthy animals. (**h**) Frequency distribution of alveolar wall thickness from cohort of healthy animals. (**i**) Absolute distribution of alveolar circularity from cohort of healthy animals. (**j**) Frequency distribution of alveolar circularity from cohort of healthy animals. n = 212 alveoli. Base, Baseline; DAPI, 4′,6-diamidino-2-phenylindole; LEA, Lycopersicon Esculentum lectin; Li, Lung Injury.

Imaging carried out over fields of view approximately 640 μm^2^ and 418 μm^2^, respectively, exposed a strong lectin signal localised to the bronchoalveolar scaffold, revealing structures including bronchioles, alveolar ducts, alveolar septa, and alveoli (Fig. 1b,c). High resolution imaging over a smaller field of view, approximately 213 μm^2^, made it possible to vividly observe an individual alveolus (Fig. 2a,b). The clarity of the individual alveoli imaged at this resolution permitted the tracing of the inner and outer wall (Fig. 2c). While the actual raw trace was utilised for all subsequent analyses, we generated a hypothetical version of these traces to facilitate a description of the analysis pipeline (Fig. 2d). To extrapolate a more accurate readout describing the thickness of the alveolar wall, we subtracted the area of the inner border (blue oval), from the area of the outer border (red oval), and expressed this as a function of the total surface area of the alveolus (red oval) (Fig. 2e). Wall thickness is expressed as a percentage of the total alveolar surface area where a smaller value represents a thinner wall.

In healthy lung tissue and in nature, alveoli are circular in shape, a geometry which reflects their capacity for expansion and contraction during respiration. To assign an objective measure to the circularity of a given alveolus we applied a shape descriptor measurement to the inner border, where an outcome 1.00 represents the perfect circle (Fig. 2f).

Imaging and analysis parameters were then applied to a cohort of alveoli from healthy lung tissue from 10 pigs (n = 212). With regard to wall thickness a normal distribution around the mean (27.37; 95% CI [26.48:28.26]) was notable on visual inspection, albeit with a skewness of 0.3749 and a kurtosis of − 0.2337 (Fig. 2g,h). In contrast circularity in this dataset exhibited a more bi-modal distribution (0.8609; 95% CI [0.8482:0.8667]) with a skewness and kurtosis of − 0.6899 and 0.02057, respectively (Fig. 2i,j). From the large sample of alveoli analysed from 10 healthy baseline pigs, we propose these data act as representative model and benchmark of alveolar wall thickness and circularity for healthy porcine lung tissue.

### Alveolar wall thickness and circularity are perturbed after gastric aspiration induced lung injury

Having established baseline values for alveolar wall thickness and circularity, we next sought to test the sensitivity of these readout parameters in detecting morphological differences in acutely injured lung tissue. To this end we performed lung tissue isolation from animals that received gastric aspiration lung injury. Injury was induced using standardized gastric contents with pH 2, delivered via a bronchoscope and monitored over 6 h while under anaesthesia.

**Figure 3:**
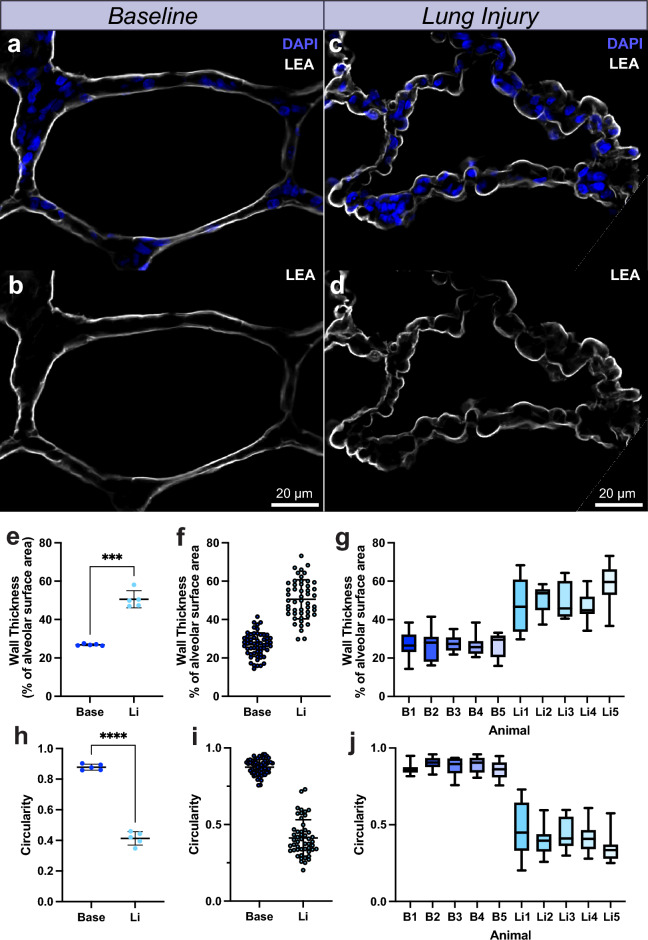
(**a**–**d**) Representative high-resolution images of single alveolus from baseline and lung injury animals (**e**) Quantification of alveolar wall thickness between baseline and lung injury animals, n = 5 animals. (**f**) Distribution of wall thickness for all alveoli analysed across 5 animals per condition, n = 50 alveoli. (**g**) Box-and-Whisker plot of alveolar thickness for each individual animal. (**h**) Quantification of alveolar circularity between baseline and lung animals, n = 5 animals. (**i**) Distribution of circularity for all alveoli analysed across 5 animals per condition, n = 50 alveoli. (**j**) Box-and-Whisker plot of alveolar circularity for each individual animal. Base, Baseline; DAPI, 4′,6-diamidino-2-phenylindole; LEA, Lycopersicon Esculentum lectin; Li, Lung Injury.*p < 0.05, **p < 0.01, ***p < 0.001, ****p < 0.0001.

Representative high resolution confocal images from single alveoli from baseline and aspiration animals exhibited noticeable differences in morphology (Fig. 3a–d). To assign objective measures to the apparent observed differences we applied our wall thickness and circularity readout to the dataset, analysing a total of 10 alveoli per animal, with 5 animals per group. Interestingly, at an animal level, alveolar wall thickness after gastric aspiration was just less than double that of baseline animals (26.86 ± 0.4998 vs 50.55 ± 4.468; p = 0.0003) (Fig. 3e). When plotting each of the individual alveoli analysed, it was also apparent that the variability in the wall thickness of gastric aspiration animals was greater (Fig. 3f). This was further validated by box-and-whisker plots for wall thickness in each individual animal (Fig. 3g). In contrast to wall thickness, alveolar circularity in the aspiration group yielded a significant decline of less than half of baseline circularity (0.8783 ± 0.01965 vs 0.4133 ± 0.04366; p < 0.0001) (Fig. 3h). As with alveolar wall thickness the variability in alveolar circularity was greater in the gastric aspiration group (Fig. 3i,j).

**Figure 4:**
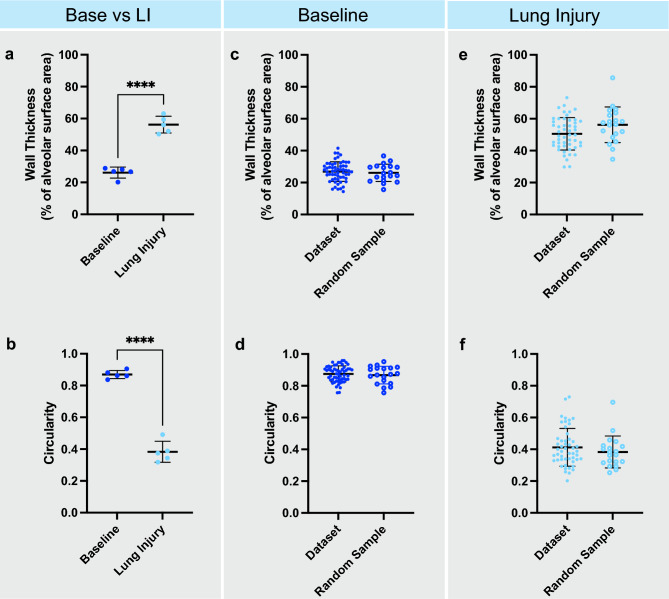
(**a**, **b**) Baseline vs lung injury comparison for alveolar wall thickness and circularity from a random sub-sample of dataset analysed by student (n = 5 animals, 4 alveoli/animal). (**c**,** d**) Comparisons of random sub-sample for baseline data for alveolar wall thickness and circularity and MQ. (**e**, **f**) Comparisons of random sub-sample for lung injury data for alveolar wall thickness and circularity.*p < 0.05, **p < 0.01, ***p < 0.001, ****p < 0.0001.

To assess the reproducibility of this technique, a researcher with no previous imaging or image analysis experience completed tracings on 4 randomly selected alveoli per animal, across baseline and ALI groups. Data from this sub-analysis revealed the same differences in wall thickness and circularity as determined by the primary analysis (Fig. 4a,b). Furthermore, comparisons between alveoli from the sub-analysis, main dataset, and within each experimental group demonstrated no differences both regarding wall thickness (Fig. 4c,d) or circularity (Fig. 4e,f).

Taken together, these data validate that our imaging and analysis pipeline can detect differences in alveolar morphology as a continuous variable, and more importantly is highly reproducible with little to no previous knowledge or skill required for reliable analytics. To validate that these analytics were actually being carried out on injured tissue, blinded scoring of H&E stained brightfield images was carried out, with ALI tissue yielding a significantly higher injury score (Fig. S1). Thus, the changes described in wall thickness and circularity do indeed represent dimensional morphological values of acute lung injury.

### Wall thickness and circularity combined as a more sensitive variable

**Figure 5:**
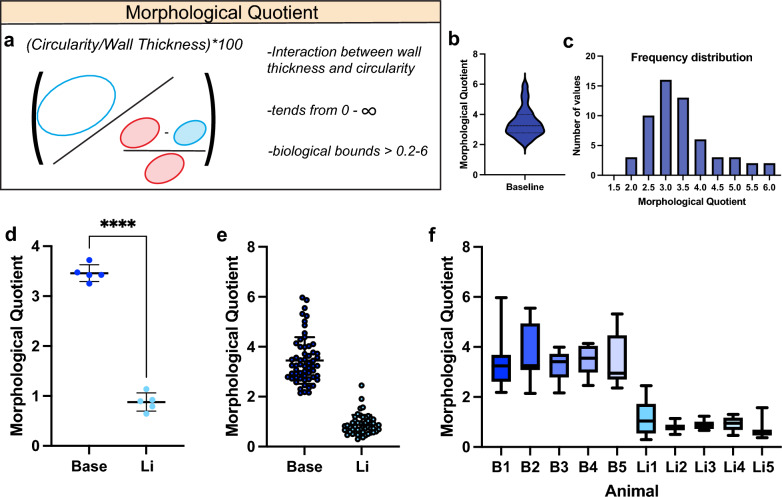
(**a**) Schematic showing how morphological quotient (MQ) is calculated. (**b**) Absolute distribution of MQ from cohort of healthy animals. (**c**) Frequency distribution of MQ from cohort of healthy animals. (**d**) Quantification of MQ between baseline and lung injury animals, n = 5 animals. (**e**) Distribution of MQ for all alveoli analysed across 5 animals per condition, n = 50 alveoli. (**f**) Box-and-Whisker plot of MQ for each individual animal. Li, Lung Injury.*p < 0.05, **p < 0.01, ***p < 0.001, ****p < 0.0001.

To infer changes in both alveolar wall thickness and circularity and their interaction utilising a single variable, we formulated a morphological quotient (MQ) where the circularity of a given alveolus is divided by its wall thickness (Fig. 5a). While the theoretical bounds of this metric span from zero to infinity, in pulmonary biology, physiological structure would place real-world bounds on this range, with a higher value representing an alveolus with both a high degree and circularity and thin wall (Fig. 5a). Applying this analysis on our original baseline dataset, we observed a normal distribution around the mean (3.345; 95% CI [3.222:3.468]) with a positive skewness (0.8590) and kurtosis (0.9140) (Fig. 5b,c).

Interestingly, when utilising this variable in the context of gastric aspiration, we uncovered a fourfold reduction compared to baseline (3.460 ± 0.1694 vs 0.8788 ± 0.1816), highlighting it as a more sensitive readout to assess overall changes in alveolar morphology (Fig. 5d,e). In addition, assessment of the MQ variable in all alveoli and individual animals analysed revealed a reduced data variance in the aspiration group compared to wall thickness and circularity variables alone (Fig. 5f). To assess the value of the MQ variable in relation to the gold standard of observer-based scoring a simple linear regression was carried out between MQ and cumulative injury score (Fig. S2). This revealed a strong negative correlation (R^2^ = 0.9919, p < 0.0001) between MQ and lung injury score, highlighting this readout as a valuable new tool to assess lung injury in a more sensitive manner.

## Discussion

To more accurately quantify and report either pathological changes that occur in a tissue due to injury or disease, or to make inferences on the capacity of a novel therapeutic to restore a tissue and its functioning to physiological levels, it is important that we establish reliable and objective readouts that possess the sensitivity to detect subtle changes.

While in the context of ALI robust standards have been put forward to outline what constitutes lung injury in animal, less focus has been afforded to the development more sophisticated readouts^6,7^. A frequent critique of animal models used to recapitulate human disease is the inability to recreate all features of ALI that would be noted in histological examination of clinical pathology samples. As a result, a readout which does not rely on the recreation of specific histological readouts, such as neutrophilic infiltration or deposition of hyaline membranes would be valuable.

One such technology and readout which we present in this work is the utilisation of a simple LEA-based fluorescent stain coupled with high resolution confocal microscopy to quantify alveolar morphology in healthy and injured lung tissue. While H&E stains imaged using a brightfield platform facilitate tissue scoring by a pathologist, the obtained images lack the detail and sensitivity provided by IF confocal imaging. The enhanced clarity and spatial resolution, along with the elimination of background signal from other tissue elements, makes it possible to develop more precise analyses. Thus, while in lung injury, histological scores are categorical, our readouts yield a continuous dataset. Based on histological scoring, for the alveolar wall to be classified as thickened, it must be at least two to four times thicker than a healthy lung sample, which is a judgment that must be made by the assessor evaluating the tissue piece ^6,7^. In contrast, our readout provides an absolute value describing the change in wall thickness, with increased resistance to variability between personnel, and reduces the need for reliable and trained scorers. Our readout can be readily recapitulated with minimal training or experience.

Although we do not seek to replace the current standards for classifying ALI, we believe that the proposed readouts outlined in this work would supplement the metrics currently accumulated on tissue morphology. Not only can our morphological assessment be used to generate continuous datasets describing the degree of alveolar perturbation in injury, but the baseline values generated from healthy lung tissue can be used going forward to benchmark how effective a therapy is in restoring normal alveolar architecture. This is especially valuable for models where biopsies can be collected at intervals across the experimental timeline from the same animal^11^. From these samples, a morphological dose/time response can be generated and compared with clinical lung function metrics.

While our alveolar wall thickness and circularity readout proved to be sensitive metrics to assess structural changes induced by lung injury induced by gastric aspiration, the addition of the MQ variable increased the sensitivity and reduced the variability in the lung injury data. This highlights MQ as a potentially valuable tool in discerning more subtle changes in alveolar morphology, which once again may be important in assessing response to treatments.

Though the step from brightfield to multiplex confocal imaging is an arduous one, this work represents a more viable transition to advanced light microscopy. This is achieved by utilising a simple and highly reproducible lectin-based stain that requires little previous experience. Interestingly, this is not the first time lectin has been used in pulmonary research, with substantial previous work cataloguing the localization of specific lectins binding in the lung. Such mapped structures include the bronchopulmonary epithelium, pulmonary blood vessels, alveolar macrophages, and mast cells ^14,19–21^.

This represents a first step in bringing a quantitaive advanced light microscopy pipeline to pre-clinical ALI research in a large animal model. The goal of this specific field is to develop therapeutics to reverse ALI, which would be valuable in particular for lung transplantation by expanding the pool of lungs that can be regenerated for use in transplantation. While this work only describes a system by which to more accurately assess alterations in alveolar morphology, future work will benefit by developing and validating multiplex high resolution immunofluorescence panels. Such pipelines will help provide insights into the molecular mechanisms of ALI, which in turn may help guide the targeted development of future treatment modalities.

## Methods

### Animals

Fifteen adult pigs (*Sus Scrofa Domesticus*) of both sexes, weighing 40–45 kg were used for the experiments. Pigs were housed in pens with a 12 h light–dark cycle, ad libitum access to water. All experimental procedures were performed according to ethical approval by the Malmö-Lund ethical Committee on Animal Research (Dnr 5.2.18-8927/16) and conducted according to the CODEX guidelines by the Swedish Research Council, Directive 2010/63/EU of the European Parliament on the protection of animals used for scientific purposes and Regulation (EU) 2019/1010 on the alignment of reporting obligations. This study complies with the ARRIVE (Animal Research: Reporting in Vivo Experiments) guidelines for reporting of animal experiments.

### Anaesthesia

Animals were premedicated with xylazine (Rompun®vet. 20 mg/mL; Bayer AG, Leverkusen, Germany; 2 mg/kg) and ketamine(Ketaminol®vet. 100 mg/mL; Farmaceutici Gellini S.p.A., Aprilia, Italy; 20 mg/kg). A peripheral intravenous (IV) line was inserted in the earlobe, and a urinary catheter was inserted into the bladder. General anaesthesia was accomplished with ketamine (Ketaminol®vet, Farmaceutici Gellini S.p.A.), midazolam (Midazolam Panpharma®,Panpharma Nordics AS, Oslo, Norway) and fentanyl (Leptanal®, Piramal CriticalCare B.V., Lilly, France) infusions. Mechanical ventilation was established using a Siemens-Elema ventilator (Servo 900 C, Siemens, Solna, Sweden) after endotracheal intubation. Ventilation with volume-controlled ventilation (VCV), a tidal volume (Vt) of 6–8 ml/kg, a peak end-expiratory pressure (PEEP) 5, an inspiratory-expiratory ratio (I:E) of 1:2, a respiratory rate (RR) of 20–25 breaths/min, and a fraction of inspired oxygen (FiO2) of 0.5. Inspiration time is set to 25% with pause time 10% to give an I:E ratio of 1:2. Ventilation was adjusted to maintain carbon dioxide levels (PaCO2) between 33 and 41 mmHg.

### Induction of lung injury

Gastric contents were collected previously from pigs deprived of food for ˃ 12 h. All animals were sedated, and an orogastric tube (36 Fr) was inserted and engaged to suction. Gastric contents collected from pigs and stored at − 80 °C and upon thawing, were strained through a paper filter (Fisher Scientific, city, country) to remove particulates, and then titrated with sterile hydrochloric acid to a standard pH of 2.00. Standardized gastric contents with pH 2 (3 ml/kg) were delivered bronchoscopically equally to each segment of the lung lobes. The gastric content was equally distributed between the different lung lobes to mimic a clinical situation of aspiration. Mechanical ventilation for 6 h followed in the VCV setting with PEEP, RR and FiO2 adjusted according to clinical demands as determined by an anesthesiologist.

### Tissue sampling and processing

Animals were sacrificed by induction of ventricular fibrillation, after which lungs were retrieved *en bloc*, and inflation with FiO2 of 0.5, ensuring no atelectasis, before staple of the trachea, as previously decribed^22^. Both baseline and injury samples were taken as wedge resections from an inflated right lower lobe. Samples were cut using surgical scissors along the wing of the lower right lobe, and were approximately 2 cm^3^ volumes (Fig. S3). For vibratome-based processing samples were immediately fixed in 4% paraformaldehyde for 48 h before transfer to 0.01% sodium azide in PBS for long term storage. 200 μm tissue sections were cut using a vibrating microtome as previously described^18^. To provide structural support for sectioning samples were embedded in 3% low melting point agarose (Sigma-Aldrich, Darmstadt, Germany) in distilled water.

For microtome-based processing samples were fixed in 4% paraformaldehyde before embedding in paraffin wax after dehydration in ethanol series. 5 µm tissue sections were cut using a microtome and stained with hematoxylin and eosin according to standard protocols.

### Fluorescent staining

Staining was carried out in 24-well plates. DAPI (1:1000) and Lycopersicon Esculentum lectin (LEA), DyLight-488 (1:500), were added to PBS. Sections were incubated in staining solution for 30 min on a rocking table and washed 3 times in PBS. Sections were mounted on Superfrost + slides with fluoromount media.

### Image acquisition

Fluorescent imaging was carried out on Nikon A1RHD confocal microscope. To capture individual alveoli a Nyquist function was employed on acquisition. All images were captured at a resolution of 1024 × 1024 pixels, 208.39 nm/pixel. Nikon files were exported as 8-bit tiff images, one per channel, for analysis. To assess baseline distributions a total of 212 alveoli were individually imaged at random locations across sections from 10 animals. For baseline versus lung injury comparisons 10 alveoli per animal (n = 5/group) were imaged at random locations across the section.

Brightfield images were captured using an Olympus light microscope where images of baseline and injured lungs were captured using 4×, 10× and 20× objectives.

### Image analysis

Fluorescence images were analysed using Fiji. A polygon selection tool was used to trace outer and inner alveolar borders. To calculate wall thickness (% of total surface area) the area of the inner border was subtracted from the outer border. Circularity metric was applied to the inner border using *shape descriptors* function. Morphological quotient (MQ) was calculated by dividing the circularity wall thickness.

Brightfield images were randomised and scored by two blinded scorers who assessed the tissue based on infiltration of inflammatory cells in the alveolar wall, invasion of inflammatory cells in the alveolar space, alveolar wall thickness, presence of proteinaceous debris and degree of haemorrhage.

### Statistics

All data were analysed using GraphPad Prism 9.

For baseline distribution assessment individual data points were plotted for wall thickness, circularity and MQ. Frequency distributions for wall thickness, circularity and MQ were plotted using respective bin sizes of 3, 0.4 and 0.5. Skewness and kurtosis were calculated for all variables. For baseline versus lung injury comparisons data were tested for normality using a Shapiro–Wilk normality test. Statistical analyses were only carried out on data at an animal level (n = 5/group). For normally distributed data with equal standard deviations an unpaired t-test was used. For normally distributed data with unequal standard deviations an unpaired t-test with Welch’s correction was used. For non-normally distributed data a Mann–Whitney test was used. Simple linear regression was used to assess the correlation between MQ and cumulative injury score. Significant data was considered as follows: *p < 0.05, **p < 0.01, ***p < 0.001, ****p < 0.0001. Figures represent mean ± SD.

### Supplementary Information

Supplementary Figures.

## Data Availability

Raw imaging datasets generated during the current study are not publicly available due to ongoing analytics development but are available from the corresponding author upon reasonable request.
