# Supplementary material for: High resolution fluorescence imaging of the alveolar scaffold as a novel tool to assess lung injury
Source: Sci Rep. 2024 Mar 20;14:6662. doi: 10.1038/s41598-024-57313-6 (PMC10954697; doi:10.1038/s41598-024-57313-6)
Supplement: Supplementary file 1 — Supplementary Figures. [file 41598_2024_57313_MOESM1_ESM.pdf]

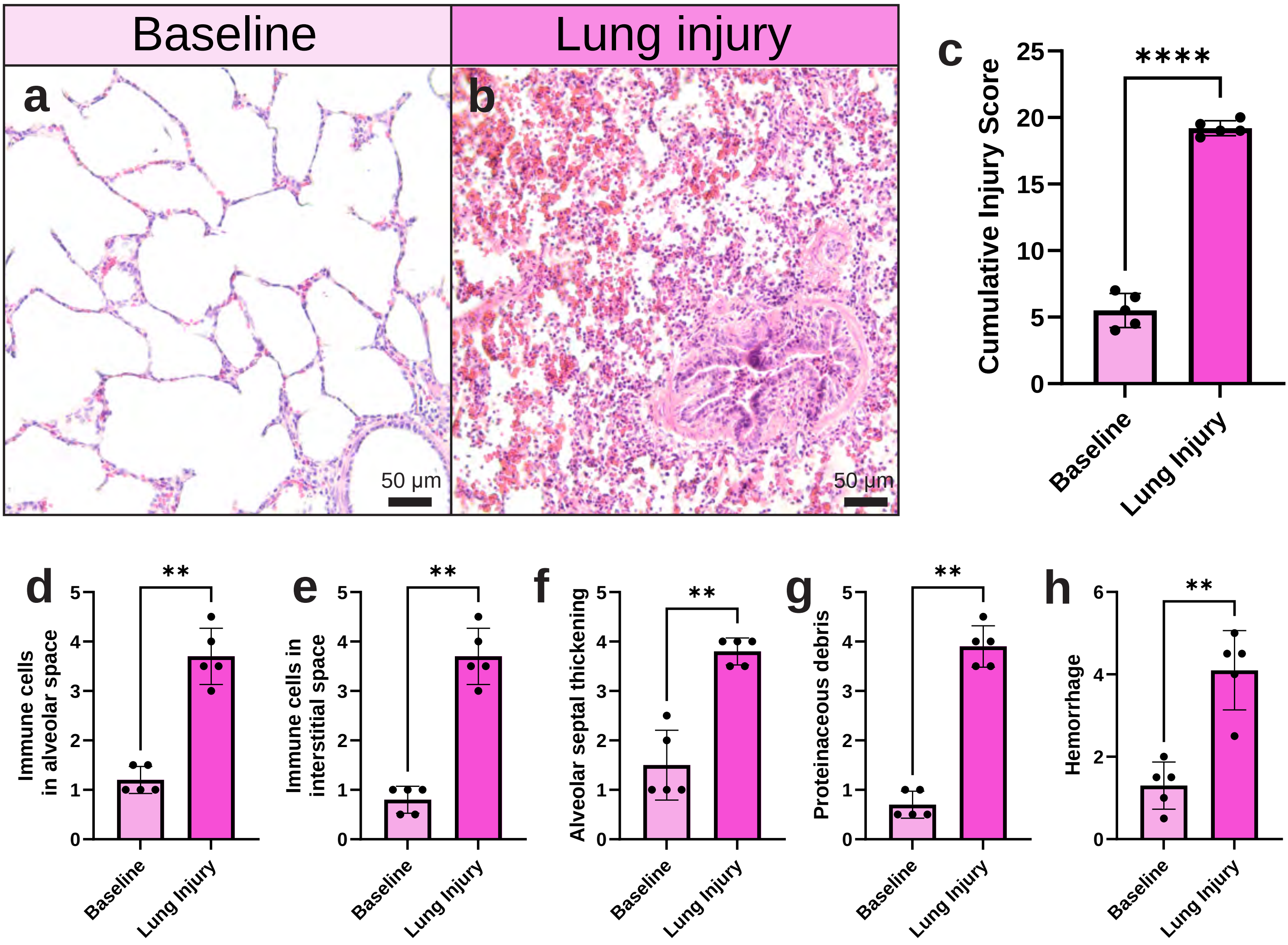

S1:

a) Representative image of baseline tissue after H&E staining/imaging. b) Representative image of injury tissue after H&E staining/imaging. c) Quantification of total injury score between baseline and lung injury tissue. d) Quantification alveolar immune cell infiltration score between baseline and lung injury tissue. e) Quantification interstitial immune cell infiltration score between baseline and lung injury tissue. f) Quantification alveolar septal thickening score between baseline and lung injury tissue. g) Quantification of degree of proteinaceous debris between baseline and lung injury tissue. h) Quantification of degree of hameorrhage between baseline and lung injury tissue. n=5 animals/group.*p < 0.05, **p < 0.01, ***p < 0.001, ****p < 0.0001.

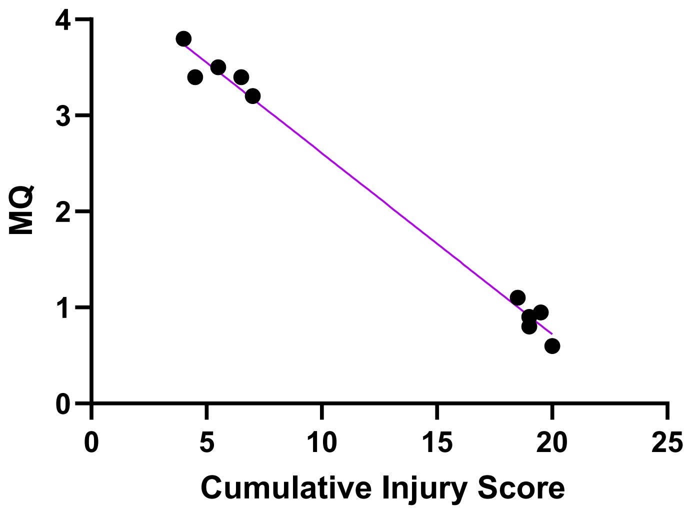

S2:
Simple linear regression to assess correlation between MQ variable and cumulative injury score. R-squared=0.9919, p<0.0001. n=10.

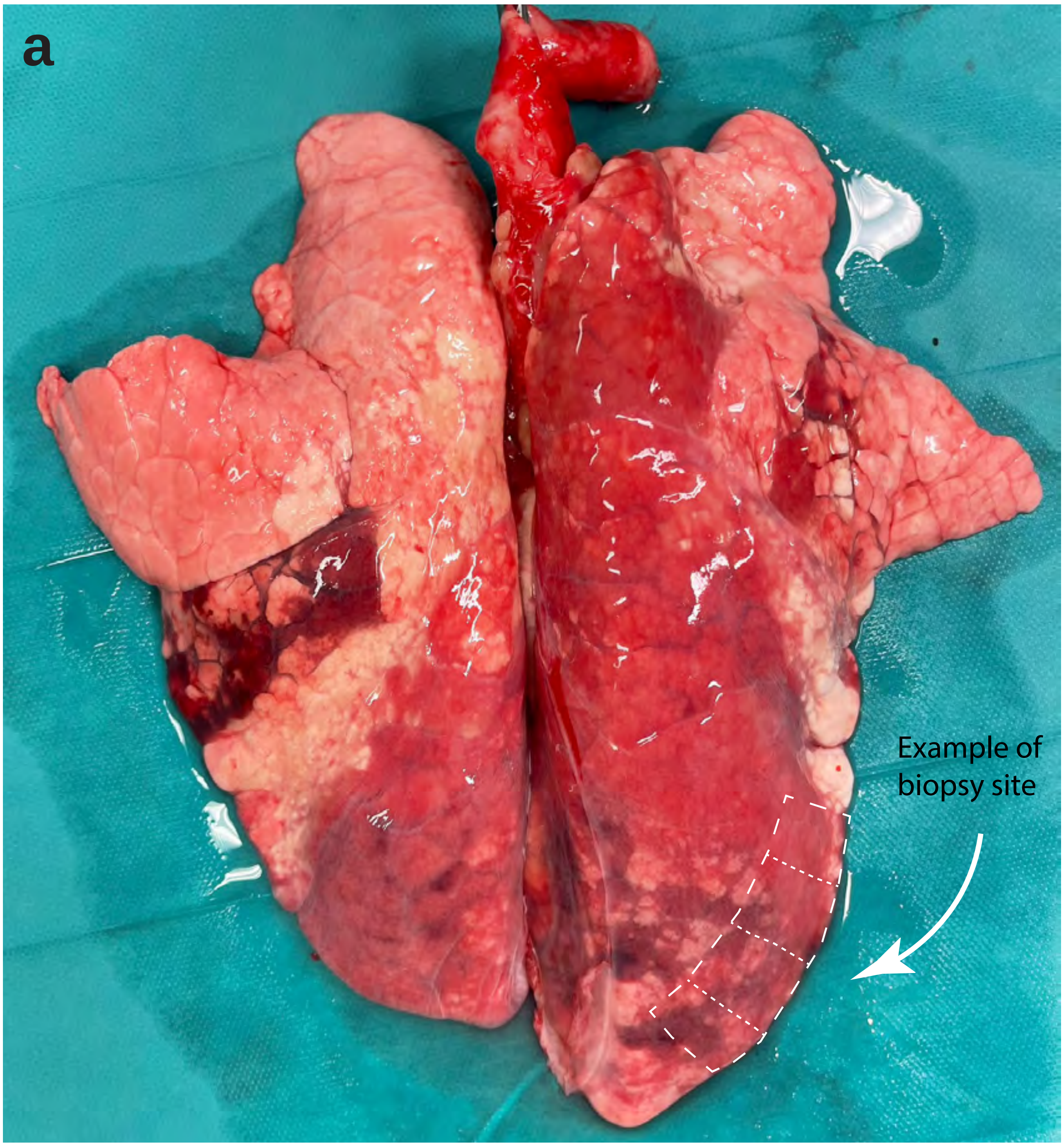

S3:
Image of lungs harvested after induction of acute lung injury wit schematic showing sample location.
